# Estimation of length-weight relationship and condition factor of spotted snakehead *Channa punctata* (Bloch) under different feeding regimes

**DOI:** 10.1186/2193-1801-2-436

**Published:** 2013-09-04

**Authors:** Surjya Narayan Datta, Vaneet Inder Kaur, Asha Dhawan, Geeta Jassal

**Affiliations:** College of Fisheries, Guru Angad Dev Veterinary and Animal Sciences University, Ludhiana, Punjab 141004 India

**Keywords:** *Channa punctata*, Formulated diets, Condition factor, Correlation coefficient, Growth

## Abstract

Comparative study was conducted to observe the efficacy of different feeding regimes on growth of *Channa punctata*. Six iso- proteinous diets were prepared by using different agro industrial by-products. Maximum weight gain was recorded with diet having 66.75% rice bran, 11.50% mustard cake, 23.0% groundnut cake, 5% molasses, 1.5% vitamin-mineral mixture and 0.5% salt with specific growth rate of 0.408. The experimental fish recorded the value of exponent ‘b’ in the range of 2.7675 to 4.3922. The condition factor ‘K’ of all experimental fish was above 1.0 (1.094- 1.235) indicating robustness or well being of experimented fish.

## Introduction

The spotted snakehead, *Channa punctata* (Bloch) is well known for its taste, high nutritive value and medicinal qualities (Haniffa *et al.*^2004^) and is recommended as a diet during convalescence (Chakraborty 2006). It is distributed throughout the South-East Asian countries and has been identified as a potential species for rearing in paddy fields, derelict and swampy water as it is an air breathing and hardy fish. It has high market value because of the flavour and availability throughout the year. The fish is suitable for both monoculture and polyculture. Good deal of work has been carried out on different aspects of survival and growth, length-weight relationship, condition factor of *C. punctata* in India and abroad (Victor and Akpocha 1992; Dutta 1994; Bias *et al.*^1994^; Alam and Parween 2001; Islam *et al.*^2004^; Kumar *et al.*^2013^). However, limited studies are conducted on growth and culture potentiality of this species. Therefore the present work has been carried out to study the efficacy of different formulated diets on survival and growth rate of *C. punctata*.

## Materials and methods

### Experimental setup

The study was conducted at the Fish Farm of College of Fisheries, Guru Angad Dev Veterinary and Animal Sciences University, Ludhiana (Punjab), India (30.54°N latitude, 75.48°E longitude and an altitude of 247 m above mean sea level). The growth of fish was assessed w.r.t. different formulated diets over a period of 90 days. The studies were conducted in PVC cistern (1.50 m×1.0 m ×1.0 m) in triplicate. 5 cm soil bed was provided in each cistern and water depth was maintained 50 cm throughout the study period. Each cistern was stocked with 25 fingerlings (average length = 11.645± 0.3145 cm, average wt. = 11.961 ± 0.1348 g) of *Channa punctata* collected from wild source.

### Formulated diets

Six isonitrogenous diets (33.19 – 35.23% crude protein on dry weight basis) i.e. D_1_, D_2_, D_3_, D_4_, D_5_ and D_6_ were formulated using agroindustrial byproducts like rice bran, mustard cake, fish meal, ground nut cake and soybean meal (Tables 1 and 2). For preparation of diets, all feed ingredients (dry) were first grounded to a small particle size in a laboratory electric grinder and sieved through an approximately 250 μm sieve. Ingredients were thoroughly mixed in a food mixer for 15 minutes. Enough water was slowly added to make stiff dough. The wet mixture was steamed for 5 minutes and the diets were produced in a noodle-like shape of 2.0 mm in diameter using a meat grinder. The pelleted diets were dried overnight at 55°C afterwards were broken up and sieved into appropriate pellet sizes. Proximate composition of feed ingredients and formulated diets was determined following the standard methods of AOAC (2005).

**Table 1:** **Percent composition of experimental diets**

Ingredients	D_1_	D_2_	D_3_	D_4_	D_5_	D_6_
Rice bran	67.11	73.15	66.75	76.49	71.86	69.29
Mustard cake	10.85	26.92	11.50	7.84	9.38	10.46
Fish meal	21.70	-	-	-	-	6.75
Groundnut cake	-	-	23.0	-	9.38	6.75
Soybean meal	-	-	-	9.38	1.88	6.75

Additives in all diets: Vitamin-mineral mixture = 1.5%, Salt = 0.5%, Molasses = 5%.

**Table 2:** **Proximate composition (% DM basis) of feed ingredients and experimental diets**

Ingredients	Moisture	Crude protein	Ether extract	Crude fibre	Ash	NFE
Rice bran	14.20	26.70	1.4	8.89	7.18	41.63
Mustard cake	13.20	57.53	1.5	7.58	7.22	12.97
Fish meal	14.65	46.80	3.0	2.99	29.76	3.25
Groundnut cake	13.95	44.59	2.5	7.81	4.21	26.94
Soybean meal	13.05	66.48	1.5	5.73	5.26	8.01
D_1_	16.00	34.22	2.66	7.68	16.12	23.32
D_2_	17.25	33.68	1.45	10.68	7.14	30.29
D_3_	17.00	34.10	1.90	9.73	5.80	30.34
D_4_	15.10	33.94	1.12	9.36	5.48	35.60
D_5_	15.15	33.74	1.75	9.49	6.13	33.94
D_6_	14.85	34.35	1.95	9.23	10.34	28.49

### Feeding of fish

Fish were fed with formulated diets @ 2% of body weight at 10 am daily. The feed quantity was regulated based on the fortnightly sampling of 10 fingerlings from each treatment.

### Water analysis

Throughout the study period physico–chemical parameters of water samples including water temperature, pH, dissolved oxygen, total alkalinity, hardness, NH_3_-N, NO_3_-N, NO_2_-N and PO_4_-P were measured following standard methods (APHA 2005).

### Growth analysis

Fish were measured in terms of weight gain and increase in length. Total length (TL) was measured to the nearest 0.1 mm using a 30 cm ruler as the distance from the tip of the anterior most part of the body to the tip of the caudal fin. Analytical balances with precision of 0.01 g were used to record body wet weight (BW).

### Following growth analysis were calculated

i. 

Where, weight recorded in gram.

ii. Length-weight relationship: The length-weight (log-transformed) relationships were determined by linear regression analysis and scatter diagrams of length and weight were plotted. The length-weight relationship of the experimented fish is worked out as per cube law given by Le Cren (1951).

Where, W=Weight of fish (g), L is observed total length (cm), ‘a’ is the regression intercept and ‘b’ is the regression slope.

The logarithmic transformation of the above formula is-

iii. Fulton’s condition factor (K): Fulton’s condition factor (K) was calculated according to Htun-Han (1978) equation as per formula given below:

Where, W=weight of fish (g), L=Length of fish (cm).

### Statistical analysis

The analysis of covariance was performed to determine variation in ‘b’ values for each species following method of Snedecor and Cochran (1967). The statistical significance of the isometric exponent (b) was analyzed by a function: ts = (b-3) / S_b_ (Sokal and Rohlf 1987), where ts is the‘t’ student statistics test value, ‘b’ is the slope and S_b_ is the standard error of ‘b’. The comparison between obtained values of t-test and the respective critical values allowed the determination of the ‘b’ values statistically significant and their inclusion in the isometric range (b=3) or allometric range (negative allometric; b<3). Statistical software SPSS 14 and PAST Ver. 1.8 used for analysing the data.

## Results and discussion

Lower dissolved oxygen content of water did not create any adverse effect on survival and growth of fish because of the accessory respiratory organ present in *Channa punctata* (Table 3). There were no significant differences in water quality parameters viz. temperature, pH, dissolved oxygen, total alkalinity, Hardness, NH_3_-N, NO_3_-N, NO_2_-N and PO_4_-P observed among different treatments and all these parameters (except dissolved oxygen content) were within the range as suggested by Boyd and Pillai (1984); Rowland (1986) and Boyd and Tucker (1998) but significant variation was observed within a single treatments in time series data of different parameters.

**Table 3:** **Water quality parameters of different treatments**

Tanks	D_1_	D_2_	D_3_	D_4_	D_5_	D_6_
Temperature °C	30.450 ± 2.717	30.612 ± 2.717	30.269 ± 2.294	29.821 ± 2.363	29.22 ± 1.683	29.672 ± 1.959
pH	8.070 ± 0.403	8.110 ± 0.428	8.204 ± 0.386	8.171 ± 0.382	8.217 ± 0.376	8.242 ± 0.369
DO (mg/l)	2.355 ± 1.316	2.202 ± 1.356	3.059 ± 1.401	2.611 ± 2.050	2.989 ± 2.187	3.469 ± 2.694
Alkalinity (mg/l)	411.090 ± 43.994	424.363 ± 43.797	363.272 ± 72.901	425.45 ± 46.79	445.09 ± 45.889	404.00 ± 36.57
Hardness (mg/l)	378.667 ± 23.626	340.000 ± 11.313	373.332 ± 26.599	372.00 ± 37.09	362.00 ± 28.33	349.33 ± 24.07
Ammonia (mg/l)	0.3591 ± 0.124	0.4245 ± 0.116	0.320 ± 0.111	0.3409 ± 0.114	0.3273 ± 0.117	0.2909 ± 0.108
Phosphate (mg/l)	1.750 ± 0.765	1.540 ± 0.745	1.654 ± 0.782	1.622 ± 0.707	1.582 ± 0.718	1.613 ± 0.773
Nitrite - NO_2_ (mg/l)	0.197 ± 0.222	0.182 ± 0.222	0.116 ± 0.222	0.114 ± 0.222	0.077 ± 0.222	0.165 ± 0.222
Nitrate - NO_3_ (mg/l)	0.308 ± 0.322	0. 340 ± 0.447	0.396 ± 0.428	0.395 ± 0. 361	0.393 ± 0.389	0.349 ± 0.406

Values are Mean ± Standard Deviation.

**Figure 1:**
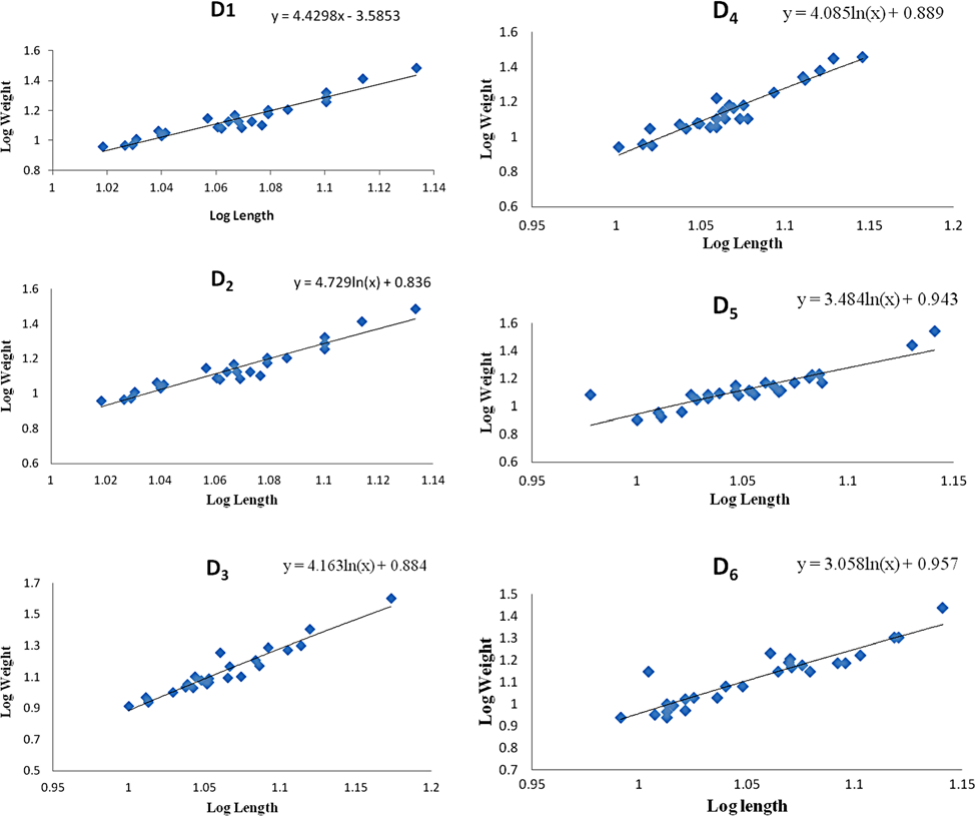
**Final logarithmic relationship between length and weight with regression equation of*Channa punctata*in experimental cysterns.**

100% survival of fish was observed in all treatments. Specific growth rate was observed maximum in D_3_ followed by D_4_, D_5_, D_2,_ D_6_ and D_1,_ respectively (Table 4). Initial and final average weight (g), Length – weight relationship of fishes stocked in different tanks, values of regression co-efficient ‘b’ and logarithmic relationship between length and weight with regression equation is given in Tables 5, 4 and Figure 1. In the present study final ‘b’ varied between 2.7675 to 4.3922. Growth is said to be positive allometric when the weight of an organism increases more than length (`>3) and negative allometric when length increases more than weight (b<3) (Wootton 1992). When TL was regressed with BW, the slope value was significantly lower than critical isometric value i.e. 3, in treatment D_1_ and D_6_ indicating negative algometric growth; thus species become slender as it increases in length (Pauly 1984) where as b value was higher than 3 in D_2_, D_3_, D_4_ and D_5_ treatment, indicating the species becomes heavier for its weight, as it grows longer (Thakur and Das 1974). The results of the present study is in conformity with the views of Le Cren (1951) and Chauhan (1987) that a fish normally does not retain the same shape or body outline throughout their lifespan and specific gravity of tissue may not remain constant, the actual relationship may depart significantly from the cube law. Negative allometric growth pattern have been reported in *C. punctata* (by Haniffa *et al.* (2006) and Ali *et al.* (2002). Negative allometric growth has also been reported in *C. maurulius* (Dua and Kumar 2006; Rathod *et al.*^2011^) and in *C. Striatus* (Khan *et al.*^2011^). Variation in slope may be attributed to sample size variation, life stages and environmental factors (Kleanthids *et al.*^1999^). The higher slope of *C. punctata* in D_2_, D_3_, D_4_ and D_5_ reflect the faster growth compared to D_1_ and D_6_ in the present study.

**Table 4:** **Final length weight relationship of fishes reared in experimental tanks**

Tank	Final average weight (g)	Specific growth rate (%/day)	Logarithmic equation Log W = log a + b log L	Correlation coefficient ‘r’	Coefficient of determination ‘r^2^’	Condition factor ‘K’	‘b’
D_1_	21.67	0.281	Log W = log 0.0151 + 2.7675 log L	0.789	0.622	1.094	2.767
D_2_	24.25	0.334	Log W = log 0.0003 + 4.3922 log L	0.930	0.865	1.116	4.392
D_3_	27.77	0.408	Log W = log 0.0011 + 3.866 log L	0.939	0.881	1.210	3.866
D_4_	25.66	0.376	Log W = log 0.0012 + 3.820 log L	0.944	0.892	1.171	3.820
D_5_	24.66	0.346	Log W = log 0.0042 + 3.3254 log L	0.876	0.768	1.334	3.325
D_6_	22.16	0.302	Log W = log 0.0118 + 2.888 log L	0.913	0.834	1.235	2.888

**Table 5:** **Initial length weight relationship of fishes reared in experimental tanks**

Tank	Initial average weight (g)	Initial logarithmic equation Log W = log a + b log L	Initial ‘b’ value
D_1_	12.09	Log W = log 0.0288 + 2.474log L	2.474
D_2_	12.13	Log W = log 0.0117 + 2.862 log L	2.862
D_3_	11.91	Log W = log 0.012 + 2.851log L	2.851
D_4_	11.76	Log W = log 0.0014 + 3.775 log L	3.775
D_5_	12.04	Log W = log 0.0104 + 2.917log L	2.917
D_6_	11.84	Log W = log 0.0107+ 2.925log L	2.925

The condition factor (K) of a fish reflects physical and biological circumstances and fluctuations by interaction among feeding conditions, parasitic infections and physiological factors (Le Cren 1951). This also indicates the changes in food reserves and therefore an indicator of the general fish condition. Moreover, body condition provides an alternative to the expensive *in vitro* proximate analyses of tissues (Sutton *et al.*^2000^). Therefore, information on condition factor can be vital to culture system management because they provide the producer with information of the specific condition under which organisms are developing (Araneda *et al.*^2008^). The values of condition factor ‘K’ recorded in the present study are 1.094, 1.116, 1.210,1.171, 1.334 and 1.235 in D_1_, D_2_, D_3_, D_4,_ D_5_ and D_6_, respectively. Condition factor of greater than one showed the well being of fishes fed with different experimental diets. The values of ‘K’ in D_2_, D_3_, D_4,_ D_5_ and D_6_ were higher than D_1_, suggesting that fish fed with diet containing different experimental diets (Table 5) were much more robust than the fish fed with diet in D_1_. The results are conformity with the study of Chandra and Jhan (2010) who recorded the K value of *Channa punctata* in the range of 1.05 – 1.89.

The co-efficient of determination (r^2^) values explained the proper fit of the model for growth. In the present study, lowest value of r^2^ of *Channa punctata* were recorded as 0.622 (62% variability) in D_1_ and highest recorded as 0.892 (89% variability) in D_4_ (Table 4) indicating more than 62% variability by the model and good fitness.

## Conclusions

In present study, growth rate, condition factor and co-efficient of determination value recorded on acclimatization of wild stock of *C. punctata* under experimental condition indicated a favourable response of the fish to the ecological transition from the wild habitat to the experimental environment. The appreciable growth rate exhibited by the fish during rearing period indicated that the prevailing environmental conditions were within the tolerance range for the species. The findings of the present study support that the species can be cultured in large scale as food fish to meet the nutritional demand.
